# The Natural Cryoprotectant Honey for Fertility Cryopreservation

**DOI:** 10.3390/bioengineering9030088

**Published:** 2022-02-22

**Authors:** Faryal Farooq Cheepa, Huilan Liu, Gang Zhao

**Affiliations:** 1 Department of Electronic Science and Technology, University of Science and Technology of China, Hefei 230027, China; faryal@mail.ustc.edu.cn; 2 Department of Blood Transfusion, First Affiliated Hospital of University of Science and Technology of China, Hefei 230001, China; huilanl@ustc.edu.cn

**Keywords:** honey, cryopreservation, extenders, natural cryoprotectant, fertility

## Abstract

Honey is a mixture of 25 sugars with other bioactive substances (i.e., organic acids, enzymes, antioxidants, and vitamins) and has been known as a highly nutritious functional food. Traditionally, it has been widely used in medicinal applications to cure various diseases. The effectiveness of honey in different applications has been used for its antimicrobial activity, absorption of hydrops, cleansing, removing odor, assisting granulation, recovery of nutrition, and formation of tissue and epithelium, which proved that honey has dehydrating and preserving properties to make it ideal for the cryopreservation of cells and tissues. Cryopreservation is an advanced preservation technique for tissue, cells, organelles, or other biological specimen storage, performed by cooling the sample at a very low temperature. It is the most common approach to improved preserving fertility (sperm, embryos, and oocytes) in different species that may undergo various life-threatening illnesses and allows for the genetic screening of these cells to test the sample for diseases before use. However, with toxic cryoprotectant (CPA), cryopreservation of fertility has been challenging because of their particular structure and sensitivity to chilling. Honey’s unique composition, as well as its dehydrating and preserving properties, qualify it to be used as a natural cryoprotectant. The aim of this study is to emphasize the ability of honey as a natural cryoprotectant in cryopreservation. The articles for this review were searched from Google Scholar, PubMed, Science Direct, Web of Science, and Scopus, using the keywords, honey, cryopreservation, natural cryoprotectant/CPAs, extenders, and fertility. Honey, as a natural cryoprotectant in fertility cryopreservation, yielded satisfactory results, with respect to improved post-thaw quality and viability. It is now proved as a non-toxic and highly efficient natural cryoprotectant in fertility preservation because its increasing viscosity at low temperature can provide a protective barrier to cells by reducing ice formation. Furthermore, its antioxidant property plays a vital role in protecting the cells from thermal damage by reducing the reactive oxygen species (ROS). This review provides a road map for future studies to investigate the potential of honey in the cryopreservation of other cells and tissue and contribute to the scientific research, regarding this remarkable natural product.

## 1. Introduction

Humans have relied on nature through many ages as a source of several different traditional medicines and for healing diseases [1]. Honey is a sweet and viscous substance, produced by bees from flower nectar or honeydew. It is greatly appreciated, not only as food, but also as medicine [2]. The use of honey in preservation is expected, as its application has a long medicinal history. It is the most persistent and oldest natural sweetening agent, and its utilization has increased immensely in the last two decades, due to its high therapeutic properties and nutritional value [3,4]. Honey rich nutritious compounds (i.e., sugars, macro and microelements, and biologically active substances) are essential for the healthy human body’s needs [5,6].

Honey has been used effectively in different applications throughout human civilization, with strong evidence. It is supposed to have started with ancient Egyptians, before 4000 BC, and was used for 30 centuries to preserve their mummies in honey [7]. It has also been used in wound healing and to treat several diseases, such as cancer, cardiovascular, ulcer, diabetic, and gastrointestinal diseases [8]. Scientific discovery in modern medicines, from time to time after the 14th century, also laid an essential foundation in the preserving procedure of honey [9].

In the 19th and early 20th centuries, some modern preservation concepts were discovered by researchers who studied freezing, cold hardiness, and freezing tolerance in the environment. This discovery is now known as “cryopreservation” [10], which usually requires cryoprotectant (CPA) to survive the impact of low-temperature freezing [11], but successful cryopreservation of biological systems is limited, due to the cytotoxicity of CPAs in both vitrification and slow freezing [12,13]. The addition of sugar in the cryoprotective freezing medium is one of the approaches to overcome the problems that limit cell viability success after thawing [14].

Due to the cryoprotectant toxicity, there is always a need for non-toxic CPAs, as an alternative to store cells at liquid nitrogen temperature; that’s why researchers recently turned back to natural material honey. Honey is now relatively widely used by researchers because it is a natural component that does not require sterilization or cause considerable side effects, which makes it the most interesting natural remedy to preserve biological cells in cryopreservation. Natural honey contains 25 sugars, mainly (fructose and glucose) comprising of about 95% of its dry weight [8,15,16]. Previous studies have proved that the cell’s survival rate has improved more effectively by the addition of two sugars mixture (sucrose and glucose) in vitrification medium, rather than the addition of sucrose alone [14]. Besides a huge portion of saccharides, many other bioactive substances are also present in honey, such as vitamins, organic acids, antioxidants, and enzymes. Such an exceptional composition of honey provides several nutritional, biological, and pharmacological effects on living cells, such as anticancer, immunosuppressive, antioxidant and antitoxin, antimicrobial, anti-inflammatory, and antimutagenic activities [8,15,16,17,18,19,20].

**Figure 1:**
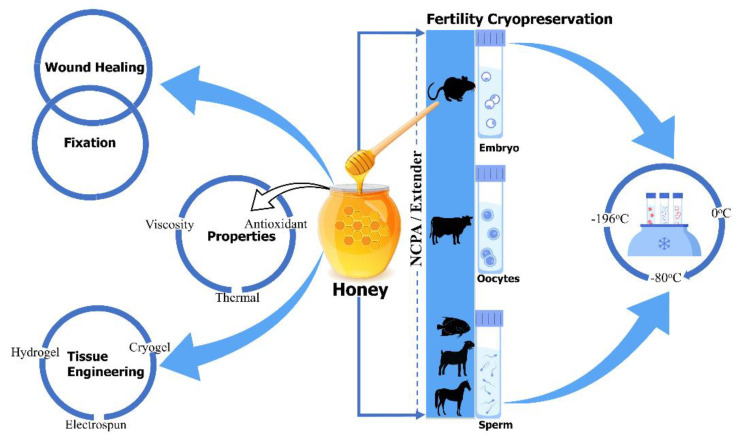
Honey’s traditional and modern application in medicine, tissue regeneration, and cryopreservation.

There are limited, but encouraging, data concerning the use of honey as a natural cryoprotectant, at most in fertility preservation, which confirmed honey’s beneficial effect on the viability of cryopreserved sperm and embryos [21,22]. Honey has been supplemented to the freezing solution of semen in goats (Maidin et al. (2018)) [23], gourami (Abinawanto et al. (2017)) [24], Arabian stallions (Reda I. El-Sheshtawy et al. (2016)) [25], and African catfish (Z.A. Muchlisin et al. (2015)) [26], aiming to improve post-thaw semen characteristics. Furthermore, Bilal Alfoteisy et al. (2020) used honey in the vitrification solution of cow bovine oocytes to improve post-thaw oocyte viability and embryonic development [27], and Fatemeh Sarmadia et al. (2019) reported that the vitrified and warmed mouse embryos with honey-based vitrification solution improved hatching and re-expansion rate of blastocyst [22]. Despite these benefits, more research must be conducted to better understand honey as a natural cryoprotectant in cryopreservation. The aim of this review was to clarify the beneficial effect of honey as a natural cryoprotectant in cryopreservation and provide a direction for future researches, in order to improve the post-thaw quality and viability of other types of cells and tissue. There is no systematic and comprehensive review focused on honey as a natural cryoprotectant. Traditional and modern applications of honey in medicine, tissue regeneration, and cryopreservation are summarized at the end of Figure 1. In this review, (1) honey and its technique to detect adulteration, (2) honey in medicinal, tissue engineering, and cryobiology application, (3) the effect of natural cryoprotectant honey on the viability of sperm, embryos, and oocytes, and, at last, (4) the current challenges and future perspectives, related to honey, are briefly discussed, in order to motivate the flourishing development of cryopreservation in the field of cryobiology.

## 2. Methods

The search methodology for data collection in this study were extracted from several online databases and search engines, including Google Scholar, PubMed, Science Direct, Web of Science, and Scopus. The inclusion criteria for articles to be considered for this review was using the following keywords, individually and in various combinations: honey, cryopreservation, natural cryoprotectant/CPAs, extenders, and fertility. The titles of articles and abstracts were considered for full-text review, and references from individual article were searched manually for relevant publications. The collected data from each article was retrieved and further verified.

## 3. Honey

Honey is one of the most used substances throughout human history, and it has always been a prized food in all parts of the world, used as a natural sweetener [28]. Furthermore, it has been traditionally used for the treatment of a variety of diseases. The specific composition of all types of honey will mainly depend on the availability of different flowers to the bees that produced the honey [29]. Honey is a supersaturated aqueous solution of inverted sugar. About 85–95% (*w*/*v*) of the total honey is comprised of saccharides and nearly 17% water [4,30]. It is a rich mixture of about 25 different sugars, which mainly consists of the monosaccharides 38% fructose and 31% glucose [31]. In addition to monosaccharides, smaller quantities of disaccharides (sucrose, galactose, alpha, beta-trehalose, gentiobiose, and laminaribiose), trisaccharides (melezitose, maltotriose, 1-ketose, panose, isomaltose glucose, erlose, isomaltotriose, theanderose, centose, isopanose, and maltopentaose), and oligosaccharides are present in honey [32]. Moreover, eighteen amino acids are found in honey. Proline was reported as the primary amino acid in honey, and it is 50–80%, compared with other types of amino acids. Proline content should be greater than 200 mg/kg. Honey is probably adulterated by sugar addition when the values are below 180 mg/kg [15,33,34,35,36]. To date, approximately 600 volatile compounds have been identified in different honey that contributes to its potential biomedical effects. It contains cyclic compounds, benzene (and its derivatives), alcohols, furan, norisoprenoids, aldehydes, acid esters, pyran, and terpene (and its derivatives), as well as sulfur, ketones, and hydrocarbons [37,38,39]. Approximately 31 different minerals have also been found in honey, including all other minerals, such as phosphorus, sodium, calcium, sulfur, magnesium, and chlorine, with potassium as the main mineral element [40]. Honey contains organic acids that are responsible for the acidic property of honey (pH between 3.2 and 4.5). Gluconic acid is the main organic acid a product of glucose oxidation present in honey; moreover, oxalic, formic, acetic, malic, butyric, citric, succinic, maleic, and lactic acids have been found [40].

Moreover, honey contains trace amounts of B vitamins, i.e., riboflavin, niacin, folic acid, pantothenic acid, and vitamin B6, as well as C vitamins, i.e., ascorbic acid. A variety of enzymes are also present in honey, such as oxidase, invertase, amylase, catalase, etc. However, glucose oxidase invertase (saccharase) and diastase (amylase) are the main enzymes in honey. They have an important role in the formation of honey (24). To make use of the miracle of honey in medicinal application on internal and external health, adulteration should be avoided.

### Adulteration Detection

An increase in the demand for honey has resulted in adulteration by direct addition of sucrose syrups that are produced from sugar beet, high-fructose corn syrup (HFCS), or maltose syrup, as well as by adding industrial sugar (glucose and fructose) syrups, obtained from starch by heat, enzyme, or acid treatment; an additional method would be by feeding the bee colonies excessively with these syrups during the main nectar period [41,42,43]. Honey adulteration affects not only honey quality, but also its production. It is essential to understand the different methods for detecting various syrup-based adulterants in honey for their authenticity before use in various applications. Fructose and glucose are the two key indicators for qualitative analysis of honey. Several new techniques have been developed during the last three decades to address specific sugar adulterants. Some techniques are specific for particular adulterants, such as when the chemical composition of the adulterant is similar to honey. The C-isotope approach is one of the oldest methods and still an effective tool to detect adulteration in honey, which relies on the carbon isotope ratio (13C/12C) differentiation between plant groups [44]. High-performance thin-layer chromatography (HPTLC) is a commonly used analytical technique [45], in addition to high-performance anion-exchange chromatography (HPAEC) [46], gas chromatography “GC” [47], and high-performance liquid chromatography (HPLC) coupled with various detectors [48]. The advancement of technology has led to the use of advanced techniques, such as infrared spectroscopy (IR) [49], nuclear magnetic resonance (NMR) [50], and Raman spectroscopy [51], each having particular advantages, concerning sample processing, before the measurement, as well as the overall measurement time. To understand the quality of honey served, one has to study its properties, which play a significant role.

## 4. Cryopreservation Related Properties

Many scientific articles have been reported, in regard to the various properties of honey. Considering the researchers’ increasing interest in using natural honey in the field of cryopreservation, we have described, in detail, some highly efficient properties that benefit from low temperature freezing, such as rheological, thermal, and antioxidants of honey.

### 4.1. Rheological Property

Rheology is the study of the flow and deformation of a material under a given pressure [2]. Viscosity is the main rheological property of honey. It is a sticky and highly viscous liquid food because of its high sugar and low water contents. In the last decade, many studies have confirmed that the viscosity of different kinds of honey is greatly influenced by temperature and water content [52,53]. The viscosity of honey usually increases with decreasing temperature and water content because of high molecular friction and greater hydrodynamic forces [54,55,56,57]. Different equations, employed in several studies, can describe the viscosity–temperature connection. While the Arrhenius model was applied widely to describe the dependence of viscosity to temperature for many types of honey, some researchers proved that this model was not appropriate for all kinds of honey [52,54,58]. More clearly, some types of honey need other models (such as William–Landel–Ferry (WLF)) to show a logical relationship between their viscosities and temperatures. To describe the dynamic viscosity of honey, the WLF model uses glass-transition temperature (Tg) and viscosity in the glass state (hg) [52,56,59,60,61].

In many publications, honey is presented as a Newtonian fluid, from the rheological viewpoint, and characterized by constant viscosity (h) at a fixed temperature, which shows a linear relationship between shear stress (s) and shear rate (g) [54,62,63,64]. However, some kinds of honey were classified as non-Newtonian fluids. Some others reported a non-Newtonian behavior for certain honey types, including pseudoplastic for Galician (Spanish honey), thixotropy for a group of karvi, heather, manuka, buckwheat, and dilatancy for eucalyptus and Nigerian honey [65,66]. For non-Newtonian fluids, the shear rate (SR) ratio defines the fluid’s apparent viscosity (happ) at a constant temperature, similar to that of Newtonian fluids; however, this coefficient changes with shear rate, while the dynamic viscosity of Newtonian fluids is shear rate independent. Additionally, a thixotropic effect has also been observed by decrease in viscosity with time, at a constant shear rate and temperature [67,68,69]. The non-Newtonian behavior may be due to high molecular compounds, such as proteins or polysaccharides (dextrans), in their compositions, which also accounts for the usually observed thixotropic property [54,70]. Additionally reported, when some paste products’ molecular weights increased, due to different physical or biochemical processes, their viscosities and elasticities changed considerably. The increasingly high viscosity of honey, during lowering of the temperature, provides a protective barrier to prevent contamination and inhibit or retard ice crystal growth on a kinetic basis. Currently, this high viscous, non-toxic solution is significantly required in the field of cryopreservation, which may allow slow permeation and perfusion into cells/tissue, to provide protective benefits.

### 4.2. Thermal Property

The thermal properties of chemicals, food, and beverages must be known to perform the various heat transfer calculations involved in designing storage and refrigeration equipment and estimating procedure time for refrigerating, freezing, warming, or drying. It strongly depends on chemical composition and temperature. The thermo-physical properties, often required for heat transfer calculations, include density, specific heat, enthalpy, thermal conductivity, and thermal diffusivity [71]. DSC analysis showed the thermal nature of the samples. Glass transition temperature (Tg) is defined when an amorphous or partially amorphous substance undergoes the transition from a glassy solid to a rubbery viscous state at a specific temperature range, defined as a single temperature. This Tg is of great significance in determining honey’s efficiency, production, thermal protection, shelf life, and stability predictability [53].

In general, honey is an over-saturated sugar solution. The two main sugars in honey are fructose and glucose, and it varies with a different type of honey. Generally, the fructose and glucose ranges are 38% and 31% [31]. The balance of these two main sugars is the significant reason for the crystallization of honey, and each relative percentage determines whether it crystallizes rapidly or slowly. The water content in honey is lower when the percentage of glucose is higher, and the crystallization will be faster. Oppositely, honey with less glucose, relative to water, is a less saturated glucose solution and slow to crystallize [72,73,74]. Therefore, one of the critical properties of a solution in freezing is its tendency towards ice formation during cooling and warming. Fatemeh et al. (in 2019) had studied the thermal behavior of honey in embryo vitrification. In this study, sucrose is replaced by natural honey in a vitrified solution, which makes it more thermodynamically favorable and reduces the chance of ice crystal formation and cryodamage [22]. Due to the high cooling and warming rates, thermal stress brings unavoidable biological responses, including an excessive production of reactive oxygen species (ROS) [75]. ROS are believed to detrimentally affect mitochondrial activities, induce apoptosis, and decrease the synthesis of adenosine triphosphate (ATP) [76,77]. It has been previously shown that the promoted accumulation of ROS in heat-shocked embryos can be undermined, at least partially, by providing exogenous antioxidants, such as melatonin, ascorbic acid, and beta-mercaptoethanol [78,79,80].

### 4.3. Antioxidant Property

The consumption of antioxidants is a basic need of everyone who wishes to live a healthy life. Its major function in cells is to eliminate the harmful free radicals produced by common metabolic processes. These elements inhibit the destructive chemical reactions that cause food spoilage and many chronic illnesses. A. Gül & T. Pehlivan, R. Khalafi et al., and M. H. Roby et al. suggested that cancer and other chronic diseases could be prevented by consuming food containing an abundance of antioxidants. The major antioxidant present in honey includes amino acids (glutamine, glutamate, glycine, aspartic acid, and threonine), phenolic compounds (phenolic acids, tocopherol, quercetin, and flavonoids), vitamin C (galagin, pinobaxin, pinocembrin, and chrisin), and enzymes (catalase and glucose oxidase) [81,82,83]. Honey is becoming a popular source of antioxidants because antioxidant supply demand has widely increased in various applications. Genetic structure disruption and cellular damage occurred, due to oxidative stress, which is caused by the shortage of balancing the chemical reactions between the production of free radicals (ROS) and the natural protective effects of the body [84,85,86]. Honey phenol quercetin content directly binds to cells and strongly inhibits cellular transcription factor activities. The transcription factors inhibition improved the process of activation and phosphorylation, which avoids the cellular effect of the free radicals. It also decreases human fibrosarcoma protein expression levels and induces apoptosis of human osteosarcoma cells [87]. Honey has been used for a long time for medical needs, but its antioxidant property have recently come into the spotlight in cryopreservation [22]. Based on these observations, Fatemeh et al. (2019) investigated honey oxidative behavior as a cryoprotectant to improve embryo vitrification. Furthermore, it substantially reduced vitrified/warmed embryos ROS levels, due to its high antioxidant property [22].

## 5. Traditional Applications of Honey

Since the earliest times, ancient Egyptians, prehistoric humans, Greece, Rome, India, and China have traditionally used honey in medicine and food [88,89]. Many prescriptions and medical indications containing honey were mentioned, in a written form, in ancient Chinese medicine (shennong), since the year 200 AD [90]. Bogdanov et al. (in 2008) claimed that the alternative branch of medicine (apitherapy) used honey bee products, including honey, pollen, propolis, and royal jelly, to improve human health at any age [91]. Natural honey has been investigated as a medicinal therapeutic agent for reproductive, neurological, oncological, and ophthalmological disorders [92].

Al-Waili and Boni, in 2003, demonstrated that honey ingestion, within a wound, could also stimulate immune responses and possesses anti-inflammatory activity [93]. It also had antineoplastic activity in experimental bladder cancer. Honey also contains antimicrobial phytochemicals that represent a rich source of leads for the development of drugs to treat microbial infections. Honey has significant positive effects on fertility [94], and it shows healthy effects on the reproductive system organs, by ameliorating altered levels of follicle-stimulating hormone (FSH), luteinizing hormone (LH), and testosterone [95].

### 5.1. Wound Healing

Recently, health specialists rediscovered honey for treating infected wounds, particularly when conventional modern therapeutic agents fail. A long time ago (2100–2000 BC), Sumerians et al. recommended honey for wound treatment [96,97]. In 384–322 BC, pale honey was stated by Aristotle as “good as a salve for sore eyes and wounds” [98,99]. The extensive research on honey’s beneficial effects in wound healing inspired A.K. Ahmed et al. to accept honey as medicine in pressure ulcers, catheter exit sites, burns, and surgical incisions healing [100]. When honey is applied to wounds, osmosis allows water to pass from wound to honey, which helps in drying the infected tissue and inhibiting bacterial, yeasts, and molds growth [101]. Even though honey absorbed water from wounds, it would still maintain a sufficiently low water activity to prevent bacterial growth [102,103]. The effective properties of honey, against the growth of bacteria, result from high sugar content, low moisture content, gluconic acid, and hydrogen peroxide [104]. Previous publications by P. Gal et al. have proven that honey efficiently clears the wound infection and protects from being infected [105].

Moreover, when honey is applied to wounds, it causes a soothing reaction and reduced burn pain rapidly [106,107]. H. English et al. reported that the honey named manuka is a gifted functional food for wound and ulcers treatment because it possesses antimicrobial activity against pathogenic bacteria (i.e., Staphylococcus aureus and Helicobacter pylori) [108]. Another study reported that manuka honey, rich in sugar content, plays an important therapeutic role in treating periodontal and gingivitis disease [109].

Honey wound application, in most clinical use, directly applies honey to the wound and covers it with gauze [110]. However, different applications required multiple dressing changes to keep the honey in the wound site, which is why this method is highly inefficient. Alternatively, tissue-engineered scaffolds have become popular as alternative vehicles for honey [111].

### 5.2. Tissue Regeneration

Tissue engineering is an emerging field that offers a broad assortment of substitutes to traditional bone graft [112]. It is concerned with the regeneration and replacement of tissues, especially with large critical wounds, where there has been a substantial loss in the skin [113,114]. Recent research using honey in tissue engineering has focused on developing tissue-engineered scaffolds to encourage wound healing [115]. These scaffolds can occur in different geometries, to efficiently treat the targeted tissue and substitute standard dressings [116]. Various scaffold fabrication techniques, including electrospinning, hydrogels, and cryogels, have become popular as vehicles for additives to improve delivery during treatment [117]. The most common appropriate scaffold types in different tissue engineering applications are electrospun, hydrogels, and cryogels because they possess different structures [118]. Adding honey to cryogels, hydrogels, and electrospun scaffolds is done to observe the scaffold geometry effect on cellular adhesion and bacterial clearance and adhesion [119].

Cryogels are the most widely applied tissue-engineered scaffolds to heal and repair skin. It is a gel matrix, formed by the cross-linkage and polymerization of various polymeric agents. The easiness of preparation, biocompatibility, and naturally interconnected macro-porosity allows for a significant potential for bone grafting application [111]. K. R. Hixon et al. and A. M. Neres Santos et al. have widely studied the use of cryogel scaffolds in biomedical applications, such as drug delivery, injectable therapeutics, wound healing, and 3D-bioprinting [111,120]. Recent research found that 5% of honey could inhibit bacterial growth, while possessing the desired porosity and mechanical properties of cryogels. Neres Santos et al. used honey on PVA or gelatin cryogels matrix for wound-healing applications [121,122]. Another study shows that the decreasing peak stress, due to the addition of honey, makes cryogels more resilient and resistive to fracture during compression [123,124].

The simple and effective electrospinning method produced thin (micro to nanoscale diameter) fibers, particularly meshes composed of nanofibers, showing amazing characteristics, for example, large surface to volume ratio (Sa/vol) and small pore size high void fraction [111,118,125]. Due to the flat geometry, electrospun scaffolds are used for wound healing applications on the skin, such as ulcers, burns, pressure, etc. [126]. Incorporating honey into electrospun nanofibers is currently the most prevalent method for utilizing honey in tissue-engineered scaffolds. Maleki et al. produced the nanofibers of honey and polyvinyl alcohol by electrospinning method and used them in wound healing. He observed that honey’s natural antibiotic properties could increase the wound dressing effectiveness by increasing the repair rate [127].

Hydrogels are nanoporous, hydrophilic gel structures, in comparison with other scaffolds. They are prepared by the crosslinking of gel precursors to form a three-dimensional network structure, which can be used in various health-related applications, such as wound dressings, food additives, cell encapsulation, tissue engineering, and drug delivery [113,128]. Mackova et al. reported that hydrogels exhibit graduated pore size, as most human body tissues have a heterogeneous morphology, which confirms the beneficial effects in tissue engineering [129].

### 5.3. Fixation

Various laboratories have proven that several carcinogenic products could be effectively replaced with honey. The preservation and protection of tissues from autolysis and putrefaction is known as fixation [130]. In the early 19th century, and even today, many researchers have observed that fixative is the major limitation of the fixation process to complete cell/tissue preservation requirements, such as physical preservation, tissue chemical properties, and autolysis prevention [131]. Researchers have suggested that honey comprises of acidic, dehydrative, and anti-bacterial properties [132]. Apart from anti-bacterial and wound healing behavior, another study focused on honey tissue hardening and anti-autolysis properties [133]. These honey properties have fixative requirements and exist in terms of a fixative, instead of a preservative. Honey is produced from many floral sources and is composed of several minerals, carbohydrates, trace elements, vitamins, ascorbic acid, and hydrogen peroxide, respectively, which are responsible for honey’s putrefactive/anti-bacterial properties. Honey maintains the quality of fixation and staining, as compared with other renowned fixatives. If methods were developed to eliminate homogenization, honey would satisfy all the properties of a fixative. Honey is the suitable alternative of many toxic fixatives and can be used as a nuclear fixative, because it is non-toxic and easily available [132].

## 6. Honey in Cryopreservation

**Figure 2:**
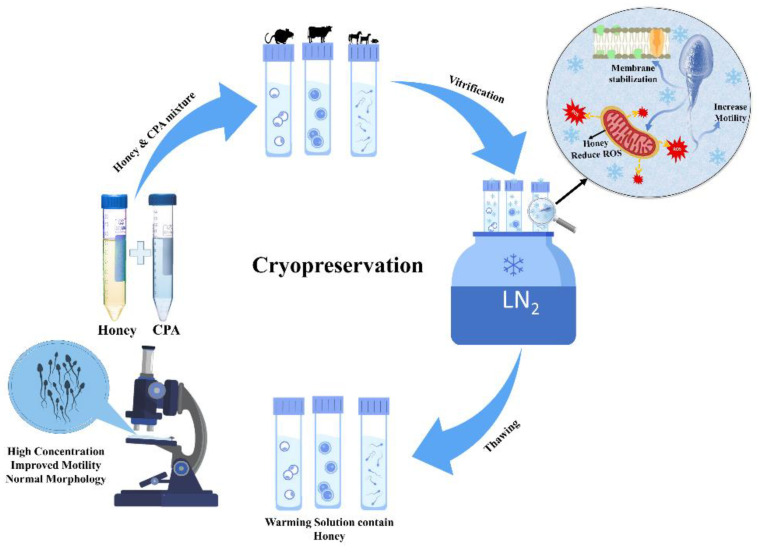
Cryopreservation of fertility, using non-permeating cryoprotectant honey during vitrification.

Since early times, it has been observed that honey can be used to preserve and protect food and tissues by several means, most notably by its osmolality and antiseptic powers, which are provided by the hydrogen peroxide and phenol content (7). However, in the early 20th century, a new concept of preservation was developed, called cryopreservation. It studies low temperature (−196 °C) to preserve living cells and tissue. With the increasing interest in using less toxic/non-toxic chemicals in cryopreservation, as a substitute for the toxic freezing solution, researchers give rise to using natural products and avoiding chemicals. Thus far, the most studied and effective use of honey in cryopreservation was extended to facilitate fertility enhancement. Moreover, recent research has revealed that honey can also act as a non-permeating cryoprotectant, due to its ice inhibition and membrane-stabilizing effect. Figure 2 shows the general process of oocytes, embryos, and sperm preservation at −196 °C.

### 6.1. Extenders

An extender is a medium to extend the volume of semen, through dilution, for artificial insemination, in order to maintain semen fertility in cryopreservation. In contrast, the addition of a freezing solution, called cryoprotectant, to the semen is used for extending the semen dilutions, which protect from cytotoxicity and osmotic stress during cryopreservation [134]. Several studies have been undertaken to use different materials and compounds, such as plant origins [135,136,137,138], whole milk, fish oil, and honey, to promote the quality of the extenders [139,140,141]. Historically, mammalian tissue fertility is enhanced by using honey in many cultures. Honey has the potential to protect the extracellular environment during cryopreservation because it contains a high amount of different sugars, which helps to increase the intracellular fluid efflux, thereby inhibiting ice crystal formation inside the cytoplasm of sperm [9,142]. Furthermore, a small amount of several antioxidant compounds are also contained in honey, including flavonoids, galagin, pinobaxin, and vitamin C [25,143].

Malik et al. discovered that the sperm motility (before freezing) and abnormality (after freezing and thawing) of semen are significantly affected by using honey to extenders. Adding honey to semen extenders noticeably improved sperm motility, acrosome integrity, membrane integrity, and viability index at 0 to 3 h of post-thawing [141]. This discovery was related to the results of other studies conducted on several species, respectively [25,144,145]. Other studies show that rosemary honey reduced DNA fragmentation, when combined with garlic and a skimmed milk-based extender [144]. The supplementation of 10% natural honey and cryoprotectant solution mixture in human semen caused a significant increase in the normal sperm morphology percentage [142,144]. Another experimental study, by El-Nattat et al., proved that different bull breed sperm quality was affected differently by adding various concentrations of honey in extenders. They suggested that 1% honey concentration, added to the extender (Bioxcell), could be more effective than Bioxcell without any additive in the cryopreservation of bull semen. For this reason, the Jersey bull showed the best quality of sperm, in comparison with other bull breeds [146,147,148]. Furthermore, El-Sheshtawy et al. (in 2016) proposed that using honey in the semen of Arab stallions, allowing for the inhibition of sperm DNA disorders, caused by oxidative stress, which protected sperm from cryoprotectant damage [21].

M. Hussain et al., J. Dorado et al., C.-H. Liu et al., and A. Ghaniei et al. have confirmed that using honey as an extender in cryopreservation improved the semen quality and acted as natural antibiotics against pathogenic bacteria. Moreover, it resolved many challenges, such as freezing solution toxicity, pH irregularity, ROS, energy source, damage of sperm membrane, and cryoshock preservatives [149,150,151,152].

### 6.2. Non-Permeating Natural Cryoprotectant

Honey is a supersaturated solution, and its unique composition provides several nutritional, pharmacological, and biological benefits to living cells, i.e., antioxidant, anti-inflammatory, antiproliferative, anticancer, antimicrobial, and antimetastatic activities [153]. It is a mixture of 25 sugars, mainly 75% monosaccharides, 10–15% disaccharides, and a minor amount of other sugars (i.e., rhamnose, erlose, trehalose, nigerobiose, sucrose, isomaltose, palatinose, maltose, maltotetraose, maltotriose, maltulose, melezitose, melibiose, raffinose, nigerose, etc.) found in honey [154,155,156,157]. It mainly consists of two kinds of sugar, fructose (38%) and glucose (31%) [31]. Besides sugars, many other bioactive substances are also present in honey, such as antioxidants, enzymes, vitamins, and organic acids [8,15,143].

Generally, sugars inhibit intracellular ice formation and prevent cell damage, due to the increasing the viscosity of the solution during vitrification, which raises the glass transition temperature to vitrify extracellular freezing solution [158]. For example, the glass transition temperature at −30 °C of sucrose solution, about 200 gL^−1^, is higher than 200 gL^−1^ of EG and glycerol solutions at −85 °C and −65 °C [159]. Sugars also act as plasma membrane stabilizing agents and protect the cell from freezing damage during cryopreservation [160]. Monosaccharides have a lower viscosity than disaccharides, which can be mixed more readily and efficiently, even in concentrated cryoprotectant solutions [161]. Disaccharides (i.e., sucrose, trehalose, and lactose) have been used in vitrification solutions as a non-permeant cryoprotectant, but commonly used disaccharides are sucrose and trehalose [162,163,164]. Raffinose (polysaccharide) has also proven to increase the survival rate of embryos after vitrification [163]. Fructose has a better effect on semen quality, as compared to disaccharides and polysaccharides, during the cryopreservation of red deer sperm [165]. Interestingly, using other disaccharides, such as sucrose and trehalose, not including lactose in dog sperm, reduces the acrosome injures and enhances the sperm viability, without any significant effect on motility after thawing, but fructose improved both the motility rates and acrosome injuries [166]. Other researchers proved that the effect of different sugars depends on their mass concentration, instead of their molar concentration during mouse sperm cryopreservation [167].

Furthermore, the supplementation of the two sugar (sucrose and glucose) mixture, instead of sucrose alone, in a vitrification solution, has more efficiently enhanced the vitrified bovine blastocysts survival rate [14]. Honey’s efficient properties, and its unique composition, make it ideal to use as natural non-permeating CPAs in cryopreservation. Table 1 summarizes the beneficial effect of using honey as a natural cryoprotectant in fertility cryopreservation. Honey decreases the effects of intracellular ice crystallization and the cytotoxic effects of CPA [168,169]. Honey is added to contribute to the vitrification medium viscosity and tonicity, which supports permeating CPAs in vitrification, which allows using lower concentrations of permeating CPAs, thus decreasing cytotoxic and osmotic shock effects permeating CPAs [170,171,172]. The honey-based media has proven to cause dehydration and rehydration in cells, sufficiently and safely, during vitrification [173,174]. In summary, the combination of honey and permeating CPAs in vitrification solution could improve mammalian tissues viability and functionalities after thawing, compared to vitrification media containing only permeating CPAs [175].

### 6.3. Fertility Cryopreservation

Cryopreservation is a fundamental tool of assisted reproduction and establishing long-term banking germplasm. Fertility cryopreservation is challenging because of gametes and embryos morphological, functional, and genetic changes in the cells after freeze- thawing [176,177,178,179]. It gives several species, suffering from various life-threatening illnesses, a chance to conceive [180,181]. Furthermore, it maximizes reproductive material availability, facilitating reproductive procedures, independent of time and geographical location [163,182].

CPAs cytotoxicity is a fundamental limiting factor for the successful cryopreservation of cells or tissue in slow freezing and vitrification [183]. The most common approach to reduce toxicity has emerged, which entails combining various cryoprotectants, both permeating and non-permeating, thereby reducing individual concentrations and mitigating damage, while maintaining the overall protective effects [184,185]. The most commonly used permeating CPAs for fertility vitrification is EG and DMSO combination. EG is being established as the primary permeating CPA, due to its low toxicity [186,187]. However, cryoprotectants have some disadvantages, in that they can induce protein denaturation at higher temperatures and cause cryoprotectant toxicity in cellular systems [188]. The addition of non-permeating CPAs sugars in cryopreservation medium is one of the approaches to overcome the problems limiting the success of cell viability after warming [174].

Notably, different saccharides in non-permeating CPAs can have different protective behavior, due to their physical and chemical properties [48]. That leads to using several sugar combinations as non-permeating CPAs to decrease cryodamage [189]. Honey is a natural compound of multiple hydrocarbons that mainly consists of fructose and glucose. It is the most commonly used sugar to preserve germplasm [190]. Furthermore, the cryoprotective performance of honey alone, or in combination with other natural cryoprotectants, has been evaluated in fertility preservation. Table 1 summarizes the selected studies for fertility cryopreservation, using a medium containing natural non-permeating cryoprotectant honey.

A few recent experiments have been conducted to evaluate the fertility of frozen germplasm (i.e., sperm, ovaries, and embryos) with honey as a natural cryoprotectant. Alfoteisy et al. (2020) conducted an experiment on cow GV-stage oocytes in honey and sucrose vitrified solution to investigate in-vitro maturation (IVM), fertilization (IVF), and embryo development (IVC). A total of 1M concentration of natural non-permeating cryoprotectant honey is suitable for bovine oocyte vitrification, as compared with sucrose. The honey group achieved an improved blastocyst (13%), as compared with sucrose (3%), and can be used in vitrification solution to improve post-warm oocyte viability and embryonic development [27]. Another fertility trial with mouse embryo in 1 Osm/L honey with EG and DMSO (h-VS2) has been conducted by Sarmadia et al. (2019). It was shown that vitrified and warmed embryos, with h-VS2 solution, showed a significantly higher hatching rate, and the re-expansion rate of vitrified blastocyst was the same as the sucrose control group. The re-expansion rate will be reduced with further increase of the concentration. The re-expansion rates with h-VS2 and the sucrose control group are 94.6% and 97.9%. The hatching rate with h-VS2 obtained significantly higher rates than the control group, from 41.9% to 43.4% [22]. Based on this data, honey can reduce the chances of cryo-damage and ice crystal formation, due to its efficient thermal and antioxidant properties. Another important observation in this experiment is the potential capability of honey as an inhibitor to ROS, thus useful in embryo cryopreservation. Putri et al. (2020) evaluated that the best concentration of honey, as a natural and extracellular cryoprotectant, is 5% to maintain sperm motility for 48 h after cryopreservation, which gave the highest motility around 87.76%, in comparison with 0% honey (69.30%) [191]. Abinawanto et al. (2017) reported that the cryopreservation of gourami spermatozoa with 0.7% of the honey solution and 10% of DMSO cryoprotectant is highly effective, which gives the highest motility (80.78%). Based on cryoprotectant activities, honey is considered as a non-permeating or extracellular cryoprotectant, while DMSO is permeating or intracellular cryoprotectant [24]. Muchlisin et al. (2015) observed the cryoprotective effect of 10% honey, compared with DMSO, to find the suitable concentration for cryopreservation of siluriformes spermatozoa [26]. Furthermore, Maidin et al. (in 2018) demonstrated that goat semen treated with honey and nigella sativa oil has higher post-thawed motility (about 60.33% at 0 and 0.5 h). In comparison with the control without supplementation, the post-thawed mobility was 24.33%, which shows that the solution could protect sperm membrane and ice formation during cryopreservation, due to oxidative stress [23]. Sheshtawy et al. (in 2016) observed significant improvement in Arab stallions on post-thawed sperm motility, viability index, membrane, and acrosome integrities at 0, 1, 2 and 3 h. Therefore, fertility preservation with natural cryoprotectant honey may have longer viability, in combination and comparison with other cryoprotectants [192].

## 7. Conclusions and Future Prospective

In conclusion, the outcomes of these studies (Table 1) evidenced that honey can provide beneficial effects in cryopreservation. If honey is used as an effective natural cryoprotectant for preserving cells and tissues in research and clinical application, it would inhibit ice crystal growth and provide a protective barrier by allowing slow perfusion and permeation into cells, due to its increasingly high viscosity at low temperatures. Additionally, its antioxidant property protects the cells from thermal damage by reducing ROS. However, different species show different tolerance ranges to the freezing-thawing process. These variations might be due to the honey concentration used in different species; therefore, the effect of various honey concentrations should be reevaluated. Overall, honey shows much potential as a nontoxic natural cryoprotectant that does not need to be removed from cells before applications in research or clinical settings. Despite all these benefits, this review can be used as a road map for future studies. More research must be conducted for a better understanding of the honey role as a cryoprotectant and contribute to the scientific research regarding this remarkable product.

## Figures and Tables

**Table 1:** Selective studies for fertility cryopreservation using natural cryoprotectant honey.

Natural CPA	Combination with Other CPAs	Cell or Tissue Type	Example of Species	Technique	Replacement Due to	Outcome	References
Natural honey	TCM-199 + EG + DMSO + CS	Bovine Oocytes	Cow	Vitrification	To investigate in vitro maturation (IVM), fertilization (IVF), and embryo development (IVC) of GV-stage oocytes vitrified in honey and sucrose solutions.	1. Natural honey acted as a non-permeating CPA in vitrification solution. 2. Improved post-warm oocyte viability and embryonic development. 3. It shows better blastocyst development than sucrose (13% vs. 3%).	Bilal Alfoteisy (2020) [27]
Natural honey	7.5% EG + 7.5% DMSO	Embryo	Mouse	Vitrification	Replace sucrose with honey to reduce the chance of ice crystal formation and cryo damage.	Natural honey makes it more thermodynamically favorable by reducing the ROS level of vitrified embryos and decreasing the chances of cryodamage.	Fatemeh Sarmadia (2019) [22]
Natural honey	Nigella sativa	Sperm	Goat	Slow freezing	Compared it with a control group without any supplement.	The combination of honey and nigella sativa gives a better effect on post-thawed sperms than fresh sperms and prevents ice crystal formation.	Maidin (2018) [23]
Natural honey	N/A	Spermatozoa	Gourami	Slow freezing	To check the suitable concentration for gourami spermatozoa.	The combination of honey and DMSO gives the highest motility in comparison with the control group (0% honey solution).	Abinawanto (2017) [24]
Natural honey	DMSO	Semen (sperm motality)	Arabian Stallion	Slow freezing	To investigate the effect of different concentrations of natural honey on post-thawed sperm motility, viability index, membrane and acrosome integrities.	Supplementation with honey (2%, 3%, and 4%) significantly improved post-thaw sperm motility, viability index. Additionally, it had a positive effect on membrane integrity and intact acrosome percentage at 0, 1, 2, 3, and 4h post-thawing.	Reda I. El-Sheshtawy (2016) [25]
Natural honey	Extender (mINRA-82 aliquots)	Sperm	African catfish	Slow freezing	To find out the cryopreservable effect of natural non-permeating cryoprotactent with frican catfish sperm, in comparison to DMSO.	A total of 10% honey allowed African catfish sperm to preserve into liquid nitrogen for 45 days.	Z.A. Muchlisin (2015) [26]
